# An Annual Exhibition for the American Medical Association

**Published:** 1872-03

**Authors:** Edward Hartshorne, D. Murray Cheston

**Affiliations:** Chairman of the Committee of Arrangements, 1439 Walnut St., Philadelphia; Secretary, 25 S. 16th St., Philadelphia


### An Annual Exhibition for the American Medical Association.

The undersigned, Chairman and Secretary of the Committee of Arrangements of the American Medical Association, have been authorized to invite attention to the project of an exhibition of objects interesting to the medical profession, to be held in Philadelphia during the next session of the Association. This exhibition has been suggested as a desirable amplification of what has been customary on these occasions; and is expected to resemble, more or less, the displays of this kind which are prominent features of the annual meetings of the British Medical Association.

In accordance with this determination to have an exhibition on the 7th, 8th, 9th and 10th of May, 1872, during the next Session of the Association, the Committee of Arrangements would respectfully and earnestly appeal for contributions of objects to be exhibited—and for other available co-operation —to members of the medical profession, pharmacists and manufacturers of chemicals, to opticians, instrument makers, publishers and booksellers, and to all others who are concerned in manufacturing or dealing in anything related to the study and practice of medicine and surgery and the associate sciences.

They will gratefully receive choice specimens and examples (likely to prove interesting through novelty, rarity, importance or superior character) of drugs, medicines, and other remedial appliances—including special chemicals and pharmaceutical compounds and materials—as well as the apparatus employed in pharmaceutical and chemical processes; also of optical and other instruments of observation and precision; of surgical instruments and implements. of preparations and objects in natural history, including human and comparative anatomy, morbid or healthy; of models, drawings, paintings, prints, and of printed works—of recent date or standard character—on medicine, surgery, and the associate sciencies.

The general plan of arrangement, and amount of space to be allotted to the several departments and collections, will have to be decided by the end of March; and all the objects to be exhibited must be within reach of the committee by the second or third week of April next.

In order, therefore to prevent confusion and disappointment, lists of objects offered for exhibition, and estimates of the amount of space desired for the purpose, will be required as soon as practicable after the publication of this circular.

Communications addressed to Wm. Pepper, M.D., 1215 Walnut street, and F. F. Maury, M. D., 1218 Walnut street, the Sub-Committee on the Exhibition, will receive immediate attention.

EDWARD HARTSHORNE, M.D.,
Chairman of the Committee of Arrangements,
1439 Walnut St., Philadelphia

[Attest, Jan. 20th, 1872.]
D. MURRAY CHESTON, M.D., Secretary,
25 S. 16th St., Philadelphia.

